# Supplementary material for: Microbiological diagnosis and mortality of tuberculosis meningitis: Systematic review and meta-analysis
Source: PLoS One. 2023 Feb 16;18(2):e0279203. doi: 10.1371/journal.pone.0279203 (PMC9934382; doi:10.1371/journal.pone.0279203)
Supplement: S2 Table — (DOCX) [file pone.0279203.s002.docx]

| S.no | Author year | Q1 | | | | Q2 | | | | Q3 | | | | Q4 | | | | Q5 | | | | Q6 | | | | Q7 | | | | Q8 | | | | Q9 | | | | Overall quality result |
| --- | --- | --- | --- | --- | --- | --- | --- | --- | --- | --- | --- | --- | --- | --- | --- | --- | --- | --- | --- | --- | --- | --- | --- | --- | --- | --- | --- | --- | --- | --- | --- | --- | --- | --- | --- | --- | --- | --- |
|  |  | Yes | No | UC | NA | Yes | No | UC | NA | Yes | No | UC | NA | Yes | No | UC | NA | Yes | No | UC | NA | Yes | No | UC | NA | Yes | No | UC | NA | Yes | No | UC | NA | Yes | No | UC | NA |  |
| 1 | Ali,et al. 2015 | / |  |  |  | / |  |  |  | / |  |  |  | / |  |  |  | / |  |  |  |  | / |  |  | / |  |  |  | / |  |  |  | / |  |  |  | 88.8% |
| 2 | Anne-Sophie, et al.2011 | / |  |  |  | / |  |  |  | / |  |  |  | / |  |  |  | / |  |  |  | / |  |  |  | / |  |  |  |  | / |  |  |  |  | / |  | 77.7% |
| 3 | Anu ,et al.2018 |  |  | / |  | / |  |  |  | / |  |  |  | / |  |  |  | / |  |  |  | / |  |  |  | / |  |  |  | / |  |  |  | / |  |  |  | 88.8% |
| 4 | Baobao,et al.2021 | / |  |  |  |  | / |  |  | / |  |  |  | / |  |  |  | / |  |  |  |  |  |  | / | / |  |  |  | / |  |  |  | / |  |  |  | 77.7% |
| 5 | Chia,et al. 2017 | / |  |  |  | / |  |  |  | / |  |  |  | / |  |  |  | / |  |  |  |  | / |  |  | / |  |  |  | / |  |  |  |  |  | / |  | 77.7% |
| 6 | Christiene,et al.2002 |  | / |  |  | / |  |  |  | / |  |  |  | / |  |  |  | / |  |  |  | / |  |  |  |  | / |  |  | / |  |  |  | / |  |  |  | 77.7% |
| 7 | Cíntia Helena,et al.2014 | / |  |  |  | / |  |  |  | / |  |  |  | / |  |  |  | / |  |  |  | / |  |  |  |  | / |  |  | / |  |  |  | / |  |  |  | 88.8% |
| 8 | Dong-Mei,et al.2020 | / |  |  |  | / |  |  |  | / |  |  |  | / |  |  |  | / |  |  |  | / |  |  |  | / |  |  |  | / |  |  |  | / |  |  |  | 100% |
| 9 | Fiona,et al.2020 | / |  |  |  | / |  |  |  |  | / |  |  | / |  |  |  | / |  |  |  | / |  |  |  | / |  |  |  | / |  |  |  | / |  |  |  | 88.8% |
| 10 | Gijs,et al.2009 | / |  |  |  | / |  |  |  |  | / |  |  | / |  |  |  | / |  |  |  | / |  |  |  |  | / |  |  | / |  |  |  | / |  |  |  | 77.7% |
| 11 | Heng ,et al.2016 | / |  |  |  | / |  |  |  | / |  |  |  | / |  |  |  | / |  |  |  | / |  |  |  | / |  |  |  | / |  |  |  | / |  |  |  | 100% |
| 12 | Hosoglu ,et al.2003 | / |  |  |  | / |  |  |  | / |  |  |  | / |  |  |  | / |  |  |  |  | / |  |  |  | / |  |  | / |  |  |  | / |  |  |  | 77.7% |
| 13 | Jaime,et al.2019 | / |  |  |  | / |  |  |  | / |  |  |  | / |  |  |  | / |  |  |  | / |  |  |  | / |  |  |  | / |  |  |  | / |  |  |  | 100% |
| 14 | Renu,et al.2017 | / |  |  |  | / |  |  |  | / |  |  |  | / |  |  |  | / |  |  |  |  |  |  | / |  | / |  |  | / |  |  |  | / |  |  |  | 88.9% |
| 15 | Robindra,et al. 2020 | / |  |  |  | / |  |  |  | / |  |  |  | / |  |  |  | / |  |  |  | / |  |  |  |  | / |  |  |  | / |  |  | / |  |  |  | 66.6% |
| 16 | Yahia,et al.2014 | / |  |  |  | / |  |  |  | / |  |  |  |  |  | / |  | / |  |  |  |  | / |  |  | / |  |  |  | / |  |  |  | / |  |  |  | 66.6% |
| 17 | Christopher,et al. 2010 | / |  |  |  | / |  |  |  | / |  |  |  | / |  |  |  | / |  |  |  |  | / |  |  | / |  |  |  | / |  |  |  | / |  |  |  | 88.8% |
| 18 | Krishnapriya,et al. 2020 | / |  |  |  | / |  |  |  | / |  |  |  | / |  |  |  | / |  |  |  | / |  |  |  |  | / |  |  | / |  |  |  | / |  |  |  | 88.8% |
| 19 | Patel ,et al.2004 | / |  |  |  | / |  |  |  | / |  |  |  | / |  |  |  | / |  |  |  |  | / |  |  | / |  |  |  | / |  |  |  | / |  |  |  | 88.8% |
| 20 | Ting,et al. 2016 | / |  |  |  | / |  |  |  | / |  |  |  | / |  |  |  | / |  |  |  | / |  |  |  | / |  |  |  |  | / |  |  |  |  | / |  | 77.7% |
| 21 | Jingya,et al.2016 |  |  | / |  | / |  |  |  | / |  |  |  | / |  |  |  | / |  |  |  | / |  |  |  | / |  |  |  | / |  |  |  | / |  |  |  | 88.8% |
| 22 | Kavitha,et al. 2016 | / |  |  |  | / |  |  |  | / |  |  |  | / |  |  |  | / |  |  |  |  |  |  | / | / |  |  |  | / |  |  |  | / |  |  |  | 88.8% |
| 23 | Duc T,et al.2019 | / |  |  |  | / |  |  |  | / |  |  |  | / |  |  |  | / |  |  |  |  | / |  |  | / |  |  |  | / |  |  |  | / |  |  |  | 88.8% |
| 24 | Egidia,et al.2015 |  | / |  |  | / |  |  |  | / |  |  |  | / |  |  |  | / |  |  |  | / |  |  |  | / |  |  |  | / |  |  |  | / |  |  |  | 88.8% |
| 25 | Erdem,et al.2013 | / |  |  |  | / |  |  |  | / |  |  |  | / |  |  |  | / |  |  |  | / |  |  |  |  | / |  |  | / |  |  |  | / |  |  |  | 88.8% |
| 26 | Filiz,et al.2011 | / |  |  |  | / |  |  |  | / |  |  |  | / |  |  |  | / |  |  |  | / |  |  |  | / |  |  |  | / |  |  |  | / |  |  |  | 100% |
| 27 | Jyothi,et al.2017 | / |  |  |  | / |  |  |  |  | / |  |  | / |  |  |  | / |  |  |  | / |  |  |  | / |  |  |  | / |  |  |  | / |  |  |  | 88.8% |
| 28 | Lidya,et al.2018 | / |  |  |  | / |  |  |  |  | / |  |  | / |  |  |  | / |  |  |  | / |  |  |  | / |  |  |  | / |  |  |  | / |  |  |  | 88.8% |
| 29 | Miguel,et al.2020 | / |  |  |  | / |  |  |  | / |  |  |  | / |  |  |  |  | / |  |  |  | / |  |  | / |  |  |  | / |  |  |  | / |  |  |  | 77.% |
| 30 | Nguyen,et al.2014 | / |  |  |  | / |  |  |  | / |  |  |  | / |  |  |  | / |  |  |  |  | / |  |  | / |  |  |  | / |  |  |  | / |  |  |  | 88.8% |
| 31 | Syed,et al.2017 | / |  |  |  | / |  |  |  | / |  |  |  | / |  |  |  | / |  |  |  | / |  |  |  | / |  |  |  | / |  |  |  | / |  |  |  | 88.8% |

Supplementary Table 2.Quality assessment of included studies
