# Supplementary material for: Microbiological diagnosis and mortality of tuberculosis meningitis: Systematic review and meta-analysis
Source: PLoS One. 2023 Feb 16;18(2):e0279203. doi: 10.1371/journal.pone.0279203 (PMC9934382; doi:10.1371/journal.pone.0279203)
Supplement: Attachment — Submitted filename: PONE-D-22-14016_R2.pdf [file pone.0279203.s007.pdf]

Abstract

Background: Tuberculosis (TB) which is caused by *Mycobacterium tuberculosis* poses a significant public health global treat. Tuberculosis meningitis (TBM) accounts for approximately 1% of all active TB cases. The diagnosis of Tuberculosis meningitis is notably difficult due to its rapid onset, nonspecific symptoms, and the difficulty of detecting *Mycobacterium tuberculosis* in cerebrospinal fluid (CSF). In 2019, 78,200 adults died of TB meningitis. This study aimed to assess the microbiological diagnosis TB meningitis using CSF and estimated the risk of death from TBM.

Methods: Relevant electronic databases and gray literature sources were searched for studies that reported presumed TBM patients. The quality of included studies was assessed using the Joanna Briggs Institute Critical Appraisal tools designed for prevalence studies. Data were summarized using Microsoft excel ver 16. The proportion of culture confirmed TBM, prevalence of drug resistance and risk of death were calculated using the random-effect model. Stata using version 16.0 was used perform the statistical analysis. Moreover, subgroup analysis was conducted. .

Results: After systematic searching and quality assessment, 31 studies were included in the final analysis. Ninety percent of the included studies were retrospective studies in design. The overall pooled estimates of CSF culture positive TBM was 29.72% (95% CI; 21.42-38.02). The pooled prevalence of MDR-TB among culture positive TBM cases was 5.19% (95% CI; 3.12-7.25). While, the proportion of INH mono-resistance was 9.37% (95% CI; 7.03-11.71). The pooled estimate of mortality rate among TBM cases was 20.42% (95%CI; 14.81-26.03). Based on sub group analysis, the pooled prevalence of mortality rate among HIV positive and HIV negative TBM individuals was 53.39% (95%CI; 40.55-66.24) and 21.65% (95%CI;4.27-39.03) respectively.

Conclusion: Definite diagnosis of TBM still remains global treat. Microbiological confirmation of TBM is not always achievable. Early microbiological confirmation of TBM has great importance to reduce mortality. There was high rate of MDR-TB among TBM patients. All TB meningitis isolates should be cultured and drug susceptibility tested using standard techniques.

Key points: Tuberculosis meningitis, microbiological diagnosis, mortality, TB culture

Introduction

Tuberculosis(TB) poses a significant public health global threat, which is caused by *Mycobacterium tuberculosis*(Mtb) bacteria. According to the World Health Organization (WHO), in 2020, the number of people newly diagnosed with TB droped to 5.8 million 10 million cases of TB were detected with 1.3 million TB deaths among HIV-negative people and an additional 214 000 among HIV-positive people (1). Following a primary or post-primary pulmonary infection, *Mycobacterium tuberculosis* can attack any part of the body including the central nervous system. Tuberculosis meningitis(TBM) is the most common type of central nervous system TB. Some patients who have or have had tuberculosis may develop the rare complication known as tuberculous meningitis. Tuberculous meningitis accounts for approximately 1 % of all cases of active tuberculosis (2).

Southeast Asia and Africa accounted for 70% of global TBM incidence. WHO estimated that 78,200 (95% UI; 52,300–104,000) adults died of TBM in 2019. Tuberculous Meningitis case fatali[illegible]n those treated was on average 27% (3, 4). Besides, TBM can cause a diverse clinical picture including altered mental status, meningitic features, seizures, cranial nerve palsies, and focal neurological deficits (5). It is among severe diseases which account 5-10% of extra-pulmonary tuberculosis cases (2).

The disease involves the infection of the meninges of the host, which is caused by Mtb and other mycobacteria. Over half of TBM survivors have neurological disability (6). Patients with TBM usually required admission to the intensive care unit. The most predisposed populations to develop TBM are children under four years, the elderly and HIV-positive patients (7). The challenge TBM management concentrated on rapid reliable diagnosis, treatment and understanding of its pathogenesis. Drug resistance and HIV infection increase the difficulty of TBM management (8).

Following TB infection infants have an up to 20% risk of developing TBM. Over half of all children with tuberculosis in the world go undiagnosed or unreported. Tuberculous meningitis mostly develops within 2–6 months following primary pulmonary infections during childhood (9). To diagnose TBM in children MRI is superior to CT imaging but its high cost and need for infrastructure make difficult to use it (10). In children, Most of the time TBM presents as

subacute meningitis which makes it difficult to distinguishes from other meningoencephalitis diseases (11).

The diagnosis of tuberculous meningitis is notably difficult due to its rapid onset, nonspecific symptom, and the difficulty of detecting *Mycobacterium tuberculosis* in cerebrospinal fluid (CSF) (12). The examination of the cerebrospinal fluid is the gold standard for diagnosing TBM. The identification of tuberculous bacilli in the CSF, either by smear examination or by culture, is required for a definitive diagnosis (13). Even though culture is the gold standard for diagnosing *Mycobacterium tuberculosis*, long time for Mycobacterium growth on Micobacterium growth indicator tube (MGIT) and LJ medium may lead to a delay in diagnosis (14).

Tuberculosis meningitis diagnosis is challenging by several factors, particularly in low- and middle-income countries: first, CSF collection necessitates lumbar puncture; second, CSF processing necessitates adequate laboratory capacity; and finally, available laboratory diagnosis methods (smear microscopy, molecular tests such as Xpert MTB/RIF, or CSF culture) have moderate sensitivity (15). A lumbar puncture is performed by a doctor who is specially trained to collect CSF. In a diagnostic Lumbar Puncture, standard bedside aseptic procedures apply with no-touch technique. At this time there were obstacles in the diagnosis of TBM due to the absence of quick, reliable and affordable diagnostic tests. This study aims to assess the microbiological diagnosis of TBM using CSF and to estimate mortality rate from TBM.

Methods

Protocol and Registration

The protocol of this systematic review and meta-analysis was registered on the PROSPERO (International Prospective Register of Systematic Reviews), University of York. It was assigned a registration number CRD42022323629 .

Literature Search

Systematic literature searching was performed using the PubMed, EMBASE databases and gray literature to assess microbiological diagnosis and mortality of Tuberculosis meningitis. The Preferred Reporting Items for Systematic Reviews and Meta-Analysis (PRISMA) checklist (16) was used to conduct this systematic review and meta-analysis (Supplementary Table 1). There was no need for ethical approval because this study was based on previously published primary

investigations. The following key terms were used to extract the intended data: Tuberculosis, meningitis, Tuberculous meningitis, diagnosis, microbiological diagnosis bacteriologically confirmed, mortality, fatality, death and TB culture .

The search terms and their variations were used in combination. The Boolean operators AND and OR were used accordingly. Articles were limited to papers published in the English language without a limit of a published year. The final search included studies published up to May 1, 2022.

Selection criteria

Included studies were: (1) original study on TBM suspected patients; (2) published in the English language without regard to a publication year; 3). having described microbiological diagnosis of tuberculous meningitis based on CSF Mycobacteriological culture result data. Additionally, included articles should be peer-reviewed, fulfilled the above listed inclusion criteria and adequately addresses the objective of the study. Studies with incomplete data, studies not used culture technique to diagnose TBM, and review articles, meta-analyses and duplicates were all excluded from the study. Two authors (GS and AA) search and select articles based on their title and abstract. Additionally, they do independent screening of the full text of the retrieved article to be included in the final analysis.

Data extraction

To collect pertinent data from each eligible study, a pre-designed Microsoft 2010 excel data extraction form was used. The extraction activity was carried out by two writers (GS and BD). The quality and completeness of the extracted data were also reviewed by the third Author (DF). The following information was extracted: initial author name; year of publication; country of study, study period, age of study participants; study design, sample size of participants, mortality rate, MDR-TB prevalence, and INH mono-resistance prevalence.

Quality Assessment

The Joanna Briggs Institute Critical Appraisal (JBI) techniques for prevalence studies were used to assess the quality of eligible papers (17). There are nine quality indicators on the JBI checklist for the prevalence study. These quality indicators were converted to 100%, and the quality score

was assessed as high if >80%, medium if 60–80%, and low if <60%. Two authors (GS and BD) carried out the quality assessment, while the third author handled the disagreement between the two authors (AA).

Data Analysis

Data were summarized and saved in Microsoft Excel 2016 before being exported to STATA Version 16.0 for analysis. All studies were pooled to estimate the risk of death of Tuberculosis meningitis suspected patients at any age. Subgroup analysis was done based on the age of study participants (children or adult), HIV status and study design. Heterogeneity among studies was examined using forest plots and I^2 heterogeneity tests. In the current review, I^2>50% a random effect model was used for analysis. Funnel plot and an Egger's test (p-value 0.1 as a significant level) to see if there was any potential for publication bias. The forest plot provides a visual inspection of the confidence intervals of effect sizes of individual studies. The presence of non-overlapping intervals suggests heterogeneity.

Result

Eligible studies

Using the study's search keyword, 1354 studies were found through a systematic search of electronic databases. After removing 1122 duplicate research, titles and abstracts were used to screen 232 publications. 174 studies were removed from the full-text review based on the abstract and title review. Only 31(18-48) papers were included in the final systematic review and meta-analysis after full-text review of 54 studies (Fig.1).

Study characteristics

There were 14 studies from Asia, eight from Europe, five from America, and only four (20, 26, 27, and 36) studies from Africa (3 in South Africa and one in Uganda) . Ninety percent of the included studies were retrospective studies in design. The study period of the studies was from 1985 to the earliest 2020. The range of sample sizes was 20(23) to 6762(36) study participants. Five studies (18, 20, 25, and 27, 32) were conducted on children under the age of 18 and seven studies were conducted on adults over the age of 18. The rest studies included all study

participants without discrimination on age. The total study participants of the included studies were 20,596 (Table 1).

Quality assessments of the included studies are provided in the supporting information (S.Table 2).Ten studies (19, 21, 22,23,28,30,33,34,38 and 47) score medium quality based on JBI quality assessment checklist for prevalence studies. While most of the studies score high quality using JBI checklist for prevalence studies.

Microbiological diagnosis

The overall pooled estimate of Tuberculosis meningitis confirmed by CSF culture was 29.72% (95 % CI; 21.42-38.02). The definite diagnosis rate was 29.72%, according to this [illegible]. The lowest percentage of TBM confirmed by CSF culture was 1.64 % (22) and the highest percentage was 85.13 % (34) (Fig.2).Prevalence of definite TBM diagnosed by AFB microscopy was 10.04% (95% CI; 4.31-15.78) (Fig 3).

Only fourteen studies reported the drug resistance pattern of the CSF culture-positive isolates. A total of 2736 CSF Mycobacterium TB culture-positive isolates were tested for drug susceptibility. Fourteen studies(5 from india,4 from china,2 from south Africa,1 from America,1 from Peru and 1 from Veitnam) were included to analyses the drug resistance pattern. MDR-TBM was found in 5.19 % of these isolates (95% CI: 3.12-7.25). (Fig.4).Eight studies reported the proportion of INH mono resistance from the above total isolates. INH mono-resistance was 9.37 % (95% CI; 7.03-11.71) (Fig.5).

Mortality rate among TBM patients

The proportion of TBM patients who died was reported in twenty-one studies. There were 1250 deaths out of a total of 6896 TBM patients. The estimated mortality rate in TBM patients was 20.42 % (95%CI; 14.81-26.03) (Fig.6).

Sub-group analysis of mortality among TBM patients

A subgroup analysis of mortality rates by age, study design type, and HIV status yields estimates of 9.80 % (95 % CI;3.22-16.37) in children under the age of 18 and 24.82 % (95 %CI;17.05-32.59) in adults (greater than or equal to 18 years old); 20.34 % (95% CI;14.03-26.65) and 30.92 % (95% CI;18.40-43.44) in retrospective and other study designs, respectively; 53.39

(95%CI;40.55-66.24) in HIV positive TBM patients and 21.65 (95%CI;4.27-39.03) among HIV negative TBM patients (Table.2).

Discussion

In this systematic review and meta-analysis the microbiological diagnosis of Tuberculosis meningitis and the risk of death among patients were calculated. According to the data around one–third of TBM patients had CSF microbiological (TB culture and AFB microscopy) confirmed illness. MDR-TB was found to be prevalent in TBM patients. The risk of death was significant among TB meningitis patients. As per the findings, one patient will die for every five TBM cases.

The culture confirmed diagnostic rate reported in this study (29.72%) was slightly near to the report (38.9%) of a previous study (49). It implies that three-fourth of TBM patients were got anti-TB treatment empirically. This finding was also in support with the reports of previous study which stated as in more than 50 per cent TBM patients, microbiological confirmation is not achieved This data indicated that conventional microbiological diagnosis of TBM tests has suboptimal positivity from CSF samples. Due to constrain of infrastructure and trained personnel, Worldwide there was a difficulty in diagnosing TBM using CSF. Junior doctors possess uncertainties regarding performing the procedure and frequently perform below expectations (50). Lumbar puncture (LP) is often not performed in sub-Saharan African and other resource-limited settings (51). Culture for *M. tuberculosis* performed on CSF had even lower positivity, producing a positive result in only approximately one in three cases (52).

Besides its longer turnaround time and inaccessibity, the lower positivity rate of CSF culture makes doubt its use as a gold standard diagnosis method for TBM. The positive rate of detection for the smear and culture tests is low alerting the globe to invest in rapid accurate and accessible diagnostic methods. Paucibacillarity of TBM makes it difficult to isolate *Mtb* in CSF by conventional culture methods. Even though rapid, sensitive and highly specific molecular detection methods have been favored, their cost and accessibility make early diagnosis of TBM difficult (53).

The lower positivity of CSF for *Mycobacterium tuberculosis* based on AF smear microscopy found in this meta-analysis was similar to other studies report which describe staining of CSF

smears for acid-fast bacilli has poor sensitivity (about 10% to 15%) (54). However, Smear microscopy is the most widely used rapid and inexpensive diagnostic test for TB, especially in low and middle-income countries. Based on this most TBM cases were not microbiologically confirmed.

This systematic review and meta-analysis study has shown that drug resistance in TBM is not an unusual occasion. The rate of MDR-TB and INH mono resistance was 5.19% and 9.37% respectively. Since most of the included studies to analyze drug resistance pattern were from Asia (5 from India, 4 from china and 1 from Vietnam), the result reflects drug resistance pattern in that specific region. This indicates that TBM has a high vulnerability to drug resistance. Thus with the difficulties of getting precious CFS samples from TBM suspected patients countries must include microbiological diagnosis of *Mycobacterium tuberculosis* in their national strategic plan and algorithm.

According to the findings, 20.03 % of TBM patients died during the course of their illness. It was consigned with the study finding of another study (55). Our sub-group analysis showed that the risk of death was higher among adults (≥18 years) and HIV positive than their respective children (<18 years old) and HIV negative patients. Majority of the included studies were done after the initiation of antiretroviral treatment in most of developed and developing countries. The different mortality rate reported in this study among children and adults was against the reports of a previous single study (56) which reports a similar 7.03% mortality rate in both groups. This finding (mortality rate among children 9.8%) is lower than the report of previous systematic review and meta-analysis (57). which reported 19.3% mortality rate among children. It might be due to the previous study participants were HIV –infected children. Among adults, our study finding was consistent with the previous studies (49, 55).

According to this study, HIV-TBM co-infected individuals have a two-fold greater mortality rate than HIV-negative patients; mortality in HIV-negative TBM patients was 21.65%, compared to 53.39 percent in HIV-positive TBM patients. A prior study (49) found a mortality rate of 53.4 percent among adult HIV-positive TBM patients, which was similar to this. The HIV-infected person is at higher risk of developing disseminated extrapulmonary tuberculosis including TBM, particularly at a stage of more advanced immunosuppression (57). It has been reported that

tuberculosis patients co-infected with HIV were more likely to have poor treatment outcomes and death (58, 59).

There was a lot of heterogeneity between studies. We were able to find subgroup analysis based on the features of the included research, but we still don't know what caused the heterogeneity. Although we were unable to pinpoint the source of heterogeneity, the following factors could contribute to publication bias and heterogeneity: 1).We only considered research that was published in English; 2).the smallest sample size of the included studies was 20; and 3).the majority of the studies were retrospective.

Our study has some limitations: First, in this meta-analysis, we only included studies published in English. Second, studies that did not use the gold standard TB culture for diagnosis were excluded. Third, due to availability of data we cannot analyse mortality by Anti-retroviral treatment usage and CD4 count. Fourth, since, there was high heterogeneity of studies interpretation of results need attention.

Conclusion

Tuberculosis meningitis cannot always be confirmed microbiologically. There was high rate of mortality in tuberculosis meningitis patients. The importance of early microbiological confirmation of TBM in reducing mortality is enormous. TBM patients have a high prevalence of MDR-TB infection. Tuberculous meningitis should be diagnosed using rapid, sensitive, and specific molecular testing methods. All TB meningitis isolates should be cultured and drug susceptibility tested using standard techniques. To investigate this goal in greater depth, prospective studies with a bigger sample size were required.

Acknowledgments

Our great acknowledge goes to the author of primary studies included in this systematic review and meta-analyses.

Conflict of Interest: The authors declare that the research was conducted in the absence of any commercial or financial relationships that could be construed as a potential conflict of interest.

Availability of Data

Data were available based on the request of the authors

Authors' contribution

GS: conceptualization, data extraction and analysis, review manuscript

AA: review manuscript, data extraction and analysis

DF: review manuscript, data extraction and analysis

BD: data extraction, review manuscript

References

1. Global tuberculosis report 2021. Geneva: World Health Organization; 2021. Licence: CC BY-NC-SA 3.0 IGO
2. Thwaites G, Fisher M, Hemingway C, et al. British Infection Society guidelines for the diagnosis and treatment of tuberculosis of the central nervous system in adults and children. *J Infect.* 2009;59(3):167–87.
3. Dodd PJ, Osman M, Cresswell FV, Stadelman AM, Lan NH, Thuong NTT, et al. The global burden of tuberculous meningitis in adults: A modelling study. *PLOS Glob Public Health* 2021;1(12).
4. Cantier M, Morisot A, Guérot E, et al. Functional outcomes in adults with tuberculous meningitis admitted to the ICU: a multicenter cohort study. *Crit Care*. 2018;22(1):1-8.)
5. Thwaites GE, van Toorn R, Schoeman J. Tuberculous meningitis: more questions, still too few answers. *Lancet Neurol*. 2013; 12: 999–1010.
6. Chiang S., Khan F, Milstein M, Tolman A.W, Benedetti A.,Starke J.R, Becerra, M.C. Treatment outcomes of childhood tuberculous meningitis: A systematic review and meta-analysis. *Lancet Infect*. Dis. 2014, 14, 947–957.
7. Cano-Portero R, Amillategui-dos Santos R, Boix-Martínez R, Larrauri-Cámara A. Epidemiology of tuberculosis in Spain. Results obtained by the National Epidemiological Surveillance Network in 2015. *Enferm Infect Microbiol Clin* 2018;36(3):179–86.

8. Faksri, K. , Prammananan, T. , Leechawengwongs, M. , Chaiprasert, A. . Molecular Epidemiology and Drug Resistance of Tuberculous Meningitis. In: Wireko-Brobby, G. , editor. Meningitis [Internet]. London: *IntechOpen*; 2012 .
9. Alarcon F, Escalante L, Perez Y, Banda H, Chacon G, Duenas G. Tuberculous meningitis. Short course of chemotherapy. *Arch Neurol*. 1990;47(12):1313–7.
10. Huynh J, Abo Y.-N, du Preez, K,Solomons R, Dooley K.E, Seddon, J.A. Tuberculous Meningitis in Children: Reducing the Burden of Death and Disability. *Pathogens* 2022, 11, 38.
11. Mezochow A, Thakur K, Vinnard C. Tuberculous meningitis in children and adults: new insights for an ancient foe. *Curr Neurol Neurosci Rep* 2017.
12. Bennett JE. Chronic meningitis. In: Bennett JE, Dolin R, Blaser MJ, eds. Mandell, Douglas, and Bennett´s. Principles and Practice of Infectious Diseases. Vol 1. 8th ed. Philadelphia, PA: Elsevier/Saunders; 2015:1138–43; ISBN 978-1-4557-4801-3
13. Ducomble T, Tolksdorf K, Karagiannis I, Hauer B, Brodhun B, Haas W, et al. The burden of extrapulmonary and meningitis tuberculosis: an investigation of national surveillance data, Germany, 2002 to 2009. *Euro Surveill* 2013; 18:.
14. van Well GT, Paes BF, Terwee CB, Springer P, Roord JJ, Donald PR, et al. Twenty years of pediatric tuberculous meningitis: a retrospective cohort study in the western cape of South Africa. *Pediatrics*. 2009;123(1).
15. Bahr NC, Meintjes G, Boulware DR. Inadequate diagnostics: the case to move beyond the bacilli for detection of meningitis due to Mycobacterium tuberculosis. *J Med Microbiol*. 2019; 68: 755–760
16. Page MJ, McKenzie JE, Bossuyt PM, Boutron I, Hoffmann TC, Mulrow CD, et al. The PRISMA 2020 statement: an updated guideline for reporting systematic reviews. *BMJ* 2021;372:n71.
17. Munn Z, Moola S, Lisy K, Riitano D, Tufanaru C. Methodological guidance for systematic reviews of observational epidemiological studies reporting prevalence and incidence data. *Int J Evid Based Healthc*. 2015;13(3):147–153.
18. Güneş A, Uluca Ü, Aktar F, Konca Ç, Şen V, Ece A, et al. Clinical, radiological and laboratory findings in 185 children with tuberculous meningitis at a single centre and relationship with the stage of the disease. *Ital J Pediatr*. 2015 Oct 15;41:75..

19. Christensen, Andersen, Å, Thomsen, V.Ø. , Andersen PH, Johansen IS. Tuberculous meningitis in Denmark: a review of 50 cases. *BMC Infect Dis* **11,** 47 (2011).
20. Goenka A, Jeena PM, Mlisana K, Solomon T, Spicer K, Stephenson R, et al. Rapid Accurate Identification of Tuberculous Meningitis Among South African Children Using a Novel Clinical Decision Tool. *Pediatr Infect Dis J*. 2018 Mar;37(3):229-234.
21. Feng, B., Fei, X., Sun, Y., Zhang X, Shang D, Zhou Y, *et al.* Prognostic factors of adult tuberculous meningitis in intensive care unit: a single-center retrospective study in East China. *BMC Neurol* 21, 308 (2021).
22. Kee CP, Periyasamy P, Law ZK, Ibrahim NM, WanYahya WNN, Mahadzir H,et al. Features and Prognostic Factors of Tuberculous Meningitis in a Tertiary Hospital in Malaysia. J *Infect Dis Epidemiol* .2017;3:028.
23. Bidstrup C, Andersen PH, Skinhøj P, Andersen ÅB. Tuberculous meningitis in a country with a low incidence of tuberculosis: still a serious disease and a diagnostic challenge. *Scandinavian Journal of Infectious Diseases*. 2002;34(11):811-4.
24. Cíntia Helena de Souza, Ayaka Yamane, Jeison Cleiton Pandini, Luciane Bisognin Ceretta, Fabiane Ferraz, Glauco Duarte da Luz ,et al. Incidence of tuberculous meningitis in the State of Santa Catarina, Brazil. Revista da Sociedade Brasileira de Medicina Tropical 47(4):483-489, Jul-Aug, 2014.
25. Wang, DM., Li, QF., Zhu, M. Wu GH, Li X, Xu YH, Zhong J, *et al.* Epidemiological, clinical characteristics and drug resistance situation of culture-confirmed children TBM in southwest of China: a 6-year retrospective study. *BMC Infect Dis* **20,** 318 (2020).
26. Cresswell FV, Tugume L, Bahr NC, Kwizera R, Bangdiwala AS, Musubire AK, et al. Xpert MTB/RIF Ultra for the diagnosis of HIV-associated tuberculous meningitis: a prospective validation study. *Lancet Infect Dis*. 2020 Mar;20(3):308-317.
27. Gijs T. J. van Well, Berbe F. Paes, Caroline B. Terwee, Priscilla Springer, John J. Roord, et al. Twenty Years of Pediatric Tuberculous Meningitis: A Retrospective Cohort Study in the Western Cape of South Africa. *Pediatrics* Volume 123, Number 1, January 2009
28. Lee, H.G., William, T., Menon, J. *et al.* Tuberculous meningitis is a major cause of mortality and morbidity in adults with central nervous system infections in Kota Kinabalu, Sabah, Malaysia: an observational study. *BMC Infect Dis* **16,** 296 (2016).

29. Hoşoğlu S, Geyik MF, Balik I, Aygen B, Erol S, Aygencel SG, et al. Tuberculous meningitis in adults in Turkey: epidemiology, diagnosis, clinic and laboratory . *Eur J Epidemiol*. 2003;18(4):337-43.
30. Soria J, Metcalf T, Mori N, Newby RE, Montano SM, Huaroto L, et al. Mortality in hospitalized patients with tuberculous meningitis. *BMC Infect Dis*. 2019 Jan 5;19(1):9.
31. Gupta R, Thakur R, Kushwaha S, Jalan N, Rawat P, Gupta P, et al. Isoniazid and rifampicin heteroresistant Mycobacterium tuberculosis isolated from tuberculous meningitis patients in India. *Indian J Tuberc*. 2018 Jan;65(1):52-56.
32. Basu Roy R, Thee S, Blázquez-Gamero D, Falcón-Neyra L, Neth O, Noguera-Julian A, et al. Performance of immune-based and microbiological tests in children with tuberculosis meningitis in Europe: a multicentre Paediatric Tuberculosis Network European Trials Group (ptbnet) study. *Eur Respir J* 2020; 56: 1902004
33. Imam YZ, Ahmedullah HS, Akhtar N, Chacko KC, Kamran S, Al Alousi F, et al. Adult tuberculous meningitis in Qatar: a descriptive retrospective study from its referral center. *Eur Neurol*. 2015;73(1-2):90-7.
34. Vinnard C, Winston CA, Wileyto EP, Macgregor RR, Bisson GP. Isoniazid resistance and death in patients with tuberculous meningitis: retrospective cohort study. *BMJ*. 2010 Sep 6;341:c4451.
35. Krishnakumariamma K, Ellappan K, Muthuraj M, Tamilarasu K, Kumar SV, Joseph NM. Molecular diagnosis, genetic diversity and drug sensitivity patterns of Mycobacterium tuberculosis strains isolated from tuberculous meningitis patients at a tertiary care hospital in South India. PLoS One. 2020 Oct 5;15(10):e0240257.
36. Patel VB, Padayatchi N, Bhigjee AI, Allen J, Bhagwan B, Moodley AA, et al. Multidrug-resistant tuberculous meningitis in KwaZulu-Natal, South Africa. *Clin Infect Dis*. 2004 Mar 15;38(6):851-6.
37. Wang, T., Feng, GD., Pang, Y., Liu JY, Zhou Y, Yang YN, et al. High rate of drug resistance among tuberculous meningitis cases in Shaanxi province, China. *Sci Rep* 6, 25251 (2016).
38. Zhang J, Hu X, Hu X, Ye Y, Shang M, An Y,et al. Clinical features, Outcomes and Molecular Profiles of Drug Resistance in Tuberculous Meningitis in non-HIV Patients. *Sci Rep*. 2016 Jan 7;6:19072.

39. Kumar K, Giribhattanavar P, Chandrashekar N, Patil S. Correlation of clinical, laboratory and drug susceptibility profiles in 176 patients with culture positive TBM in a tertiary neurocare centre. *Diagn Microbiol Infect Dis*. 2016 Dec;86(4):372-376.
40. Nguyen DT, Agarwal S, Graviss EA .Trends of tuberculosis meningitis and associated mortality in Texas, 2010-2017, a large population based analysis. *PLoS ONE* 2019;14(2): e0212729
41. Miftode EG, Dorneanu OS, Leca DA, Juganariu G, Teodor A, Hurmuzache M, et al. Tuberculous Meningitis in Children and Adults: A 10- Year Retrospective Comparative Analysis. *PLoS ONE* .2015;10(7): e0133477
42. Erdem H, Ozturk-Engin D, Elaldi N, Gulsun S, Sengoz G, Crisan A, et al. The microbiological diagnosis of tuberculous meningitis: results of Haydarpasa-1 study. *Clin Microbiol Infect*. 2014 Oct;20(10):O600-8.
43. Pehlivanoglu F, Yasar KK, Sengoz G. Tuberculous meningitis in adults: a review of 160 cases. *ScientificWorldJournal*. 2012;2012:169028.
44. Kaviyil JE, Ravikumar R. Diagnosis of tuberculous meningitis: Current scenario from a Tertiary Neurocare Centre in India. *Indian J Tuberc.* (2017)
45. Chaidir L, Annisa J, Dian S, Parwati I, Alisjahbana A, Purnama F, et al. Microbiological diagnosis of adult tuberculous meningitis in a ten-year cohort in Indonesia. *Diagn Microbiol Infect Dis.* 2018 May;91(1):42-46.
46. García-Grimshaw M, Gutiérrez-Manjarrez FA, Navarro-Álvarez S, González-Duarte A. Clinical, Imaging, and Laboratory Characteristics of Adult Mexican Patients with Tuberculous Meningitis: A Retrospective Cohort Study. *J Epidemiol Glob Health*. 2020 Mar;10(1):59-64.
47. Nhu NT, Heemskerk D, Thu do DA, Chau TT, Mai NT, Nghia HD, et al. Evaluation of GeneXpert MTB/RIF for diagnosis of tuberculous meningitis. *J Clin Microbiol.* 2014 Jan;52(1):226-33.
48. Rufai SB, Singh A, Singh J, Kumar P, Sankar MM, Singh S.. Diagnostic usefulness of Xpert MTB/RIF assay for detection of tuberculous meningitis using cerebrospinal fluid. *J Infect*. 2017 Aug;75(2):125-131.

49. Ming-Gui Wang , Lan Luo , Yunxia Zhang, Xiangming Liu, Lin Liu and Jian-Qing He. Treatment outcomes of tuberculous meningitis in adults: a systematic review and meta-analysis. *BMC Pulmonary Medicine* (2019) 19:200.
50. Henriksen MJV, Wienecke T, Thagesen H, Jacobsen RVB, Subhi Y, Ringsted C, Konge L. Assessment of Residents Readiness to Perform Lumbar Puncture: A Validation Study. *J Gen Intern Med.* 2017 Jun;32(6):610-618.
51. Siddiqi OK, Birbeck GL, Ghebremichael M, Mubanga E, Love S, Buback C, et al. Prospective Cohort Study on Performance of Cerebrospinal Fluid (CSF) Xpert MTB/RIF, CSF Lipoarabinomannan (LAM) Lateral Flow Assay (LFA), and Urine LAM LFA for Diagnosis of Tuberculous Meningitis in Zambia. *J Clin Microbiol*. 2019 Jul 26;57(8):e00652-19.
52. Garg RK. Microbiological diagnosis of tuberculous meningitis: Phenotype to genotype. *Indian J Med Res.* 2019 Nov;150(5):448-457
53. Marais S, Thwaites G, Schoeman JF, Toeroek ME, Misra UK, Prasad K, et al. Tuberculous meningitis: a uniform case definition for use in clinical research. *Lancet Infect Dis*. 2010;10(11):803 –12
54. Bahr NC, Meintjes G, Boulware DR. 2019. Inadequate diagnostics: the case to move beyond the bacilli for detection of meningitis due to Mycobacterium tuberculosis. *J Med Microbiol* 68:755–760.
55. Stadelman AM, Ellis J, Samuels THA, Mutengesa E, Dobbin J, Ssebambulidde K, et al. Treatment Outcomes in Adult Tuberculous Meningitis: A Systematic Review and Meta-analysis. *Open Forum Infect Dis.* 2020 Jun 30;7(8):ofaa257.
56. Miftode EG, Dorneanu OS, Leca DA, Juganariu G, Teodor A, Hurmuzache M,et al. Tuberculous Meningitis in Children and Adults: A 10-Year Retrospective Comparative Analysis. *PLoS One.* 2015 Jul 17;10(7):e0133477.
57. Vinnard C, Macgregor RR. Tuberculous meningitis in HIV-infected individuals. *Curr HIV/AIDS Rep.* 2009 Aug;6(3):139-45)
58. Tavares AM, Fronteira I, Couto I, Machado D, Viveiros M, Abecasis AB, et al. HIV and tuberculosis co-infection among migrants in Europe: a systematic review on the prevalence, incidence and mortality. *PLoS One*. 2017;12(9): e0185526. 35.

59. Gupta RK, Lucas SB, Fielding KL, Lawn SD. Prevalence of tuberculosis in post-mortem studies of HIV-infected adults and children in resource-limited settings: a systematic review and meta-analysis. AIDS. 2015;29(15):1987 – 2002.

Figures

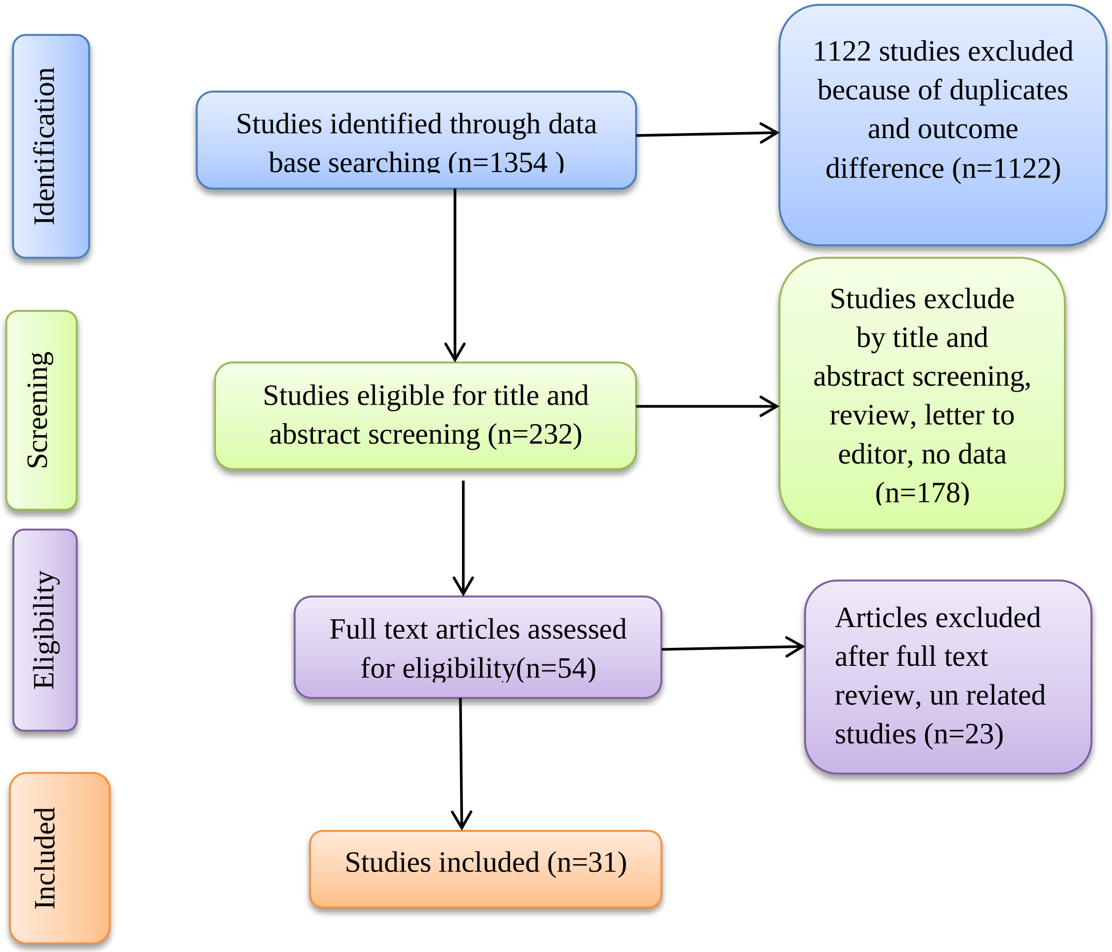

Figure.1 Flow diagram of systematic search of studies for this systematic review and meta-analysis.

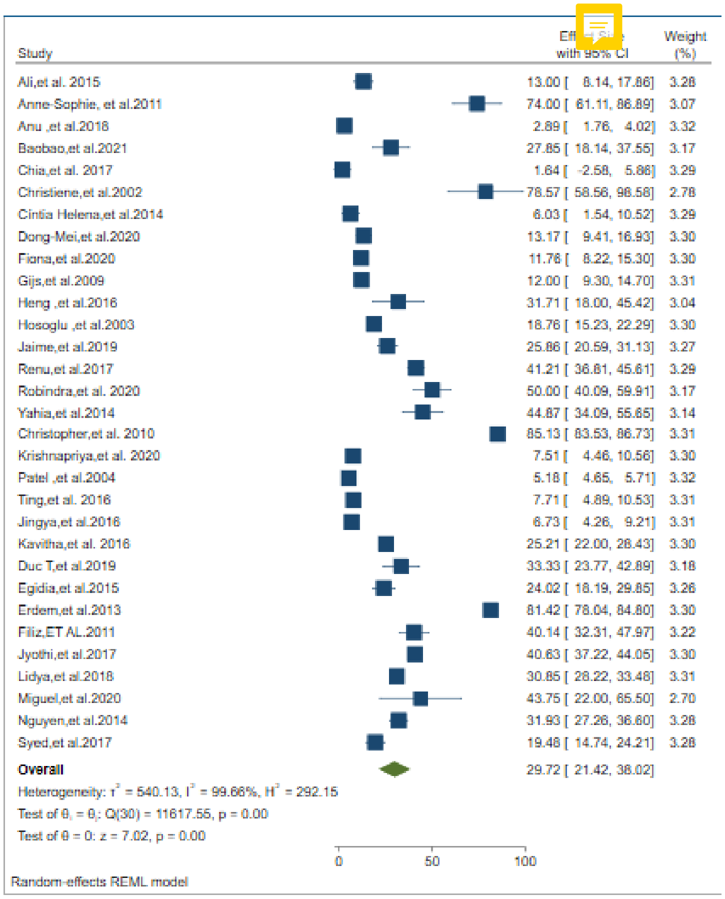

Figure.2 CSF Culture confirmed Tuberculosis meningitis among suspected patients

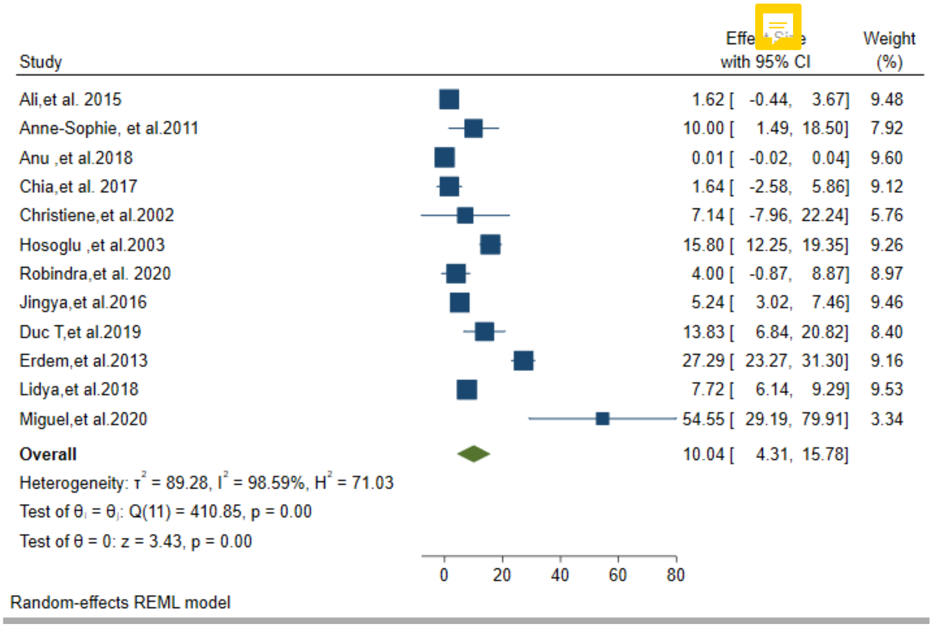

Figure.3 ZN AFB microscopy positivity of CSF in TBM suspected patients

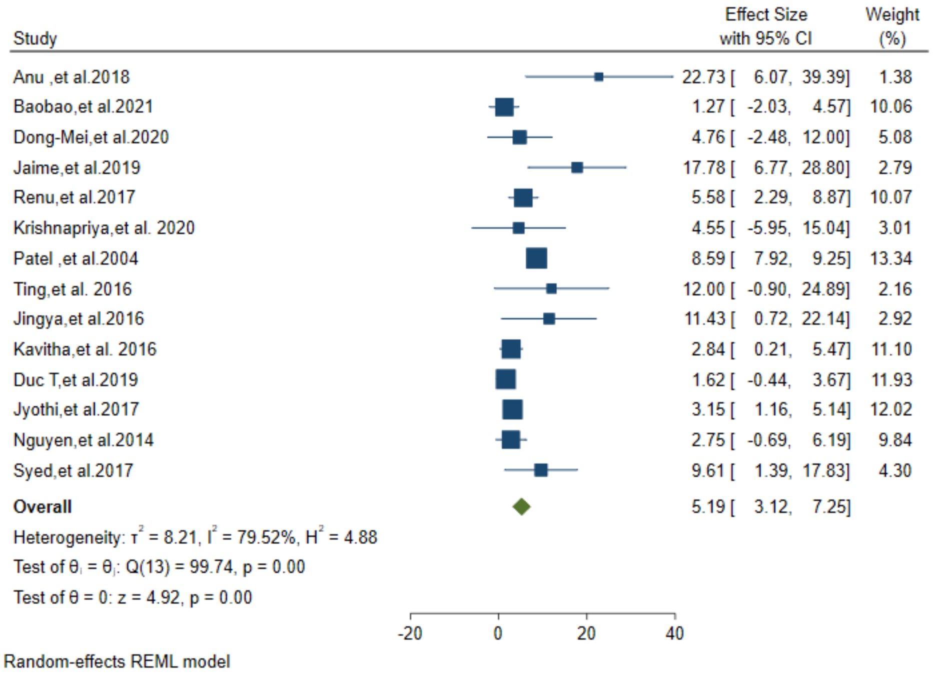

Figure.4 Pooled estimate of MDR-TB prevalence in Tuberculosis confirmed isolates.

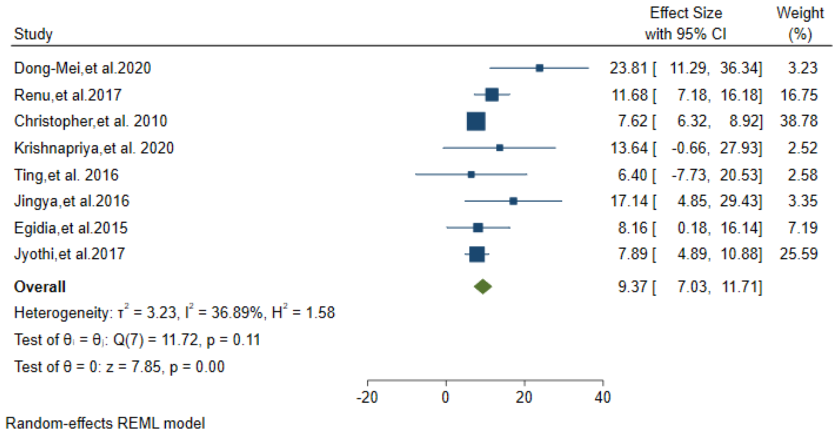

Figure.5 prevalence of INH mono resistance in Tuberculosis meningitis confirmed isolates.

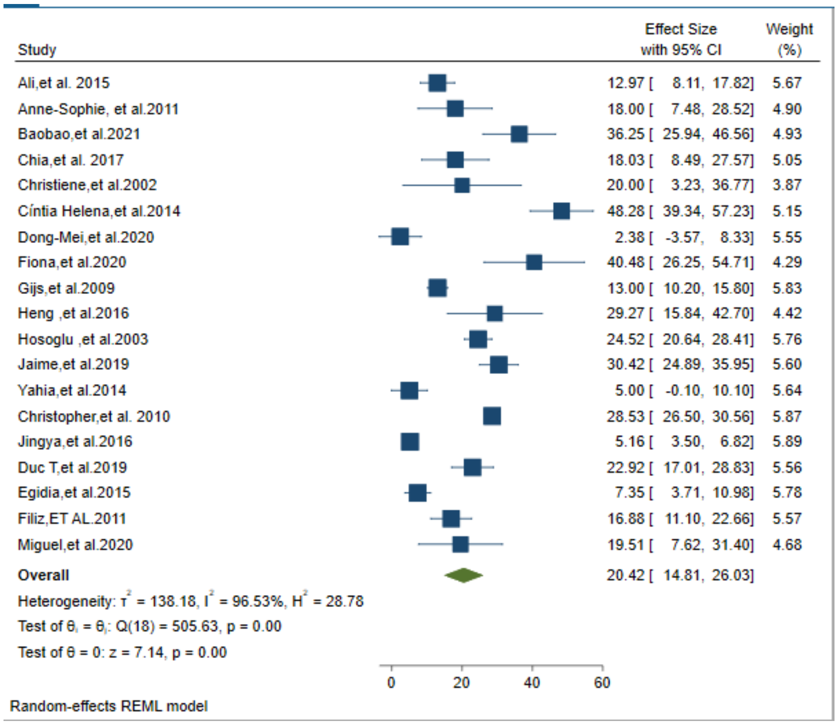

Figure.6 Mortality among Tuberculosis meningitis suspected patients.

Table 1.Study characteristic of included studies.

Author_year	Country	Study period	Study design	Participant age	Sample size
Ali,et al. 2015(18)	Diyarbakir Turkey	1998 to 2008	Retrospective	<18	185 TBM
Anne-Sophie, et al.2011(19)	Denmark	January 2000 to December 2008	Retrospective	All age	50 TBM
Anu ,et al.2018(20)	S/Africa	2010-2014	Retrospective	3 months-15 years	865 TBM
Baobao,et al.2021(21)	Shandong , China	January 2008 to April 2018.	Retrospective	>18	80 TBM
Chia,et al. 2017(22)	Kebangsaan Malaysia	January 2003 to February 2015	Observational	>18	61 TBM
Christiene,et al.2002(23)	Denmark	1988 to July 2000.	Retrospective	All age	20 TBM
Cíntia Helena,et al.2014(24)	Brazil	2001 to 2010	Descriptive	All age	116 TBM
Dong-Mei,et al.2020(25)	Southwest of China	January 2013 to December 2018	Retrospective	< 14 years old	319 TBM
Fiona,et al.2020(26)	Uganda	Nov 25, 2016, to Jan 24, 2019	Retrospective	>18	204TBM
Gijs,et al.2009(27)	South Africa	January 1985 to April 2005	Retrospective	<18	554TBM
Heng ,et al.2016(28)	Sabah, Malaysia	February 2012 to March 2013	cohort	>12	84 TBM
Hosoglu ,et al.2003(29)	Turkey	1985 to 1998	Retrospective	>18	469TBM
Jaime,et al.2019(30)	Peru	2006 to 2015	Retrospective	>18	263TBM
Renu,et al.2017(31)	India	July 2012 to July 2015	Prospective	All age	197 TBM
Robindra,et al. 2020(32)	Europe	February 2016 to August 2016	Retrospective	0–16 years	118 TBM
Yahia,et al.2014(33)	Qatar	January 2006 to December 2012	Retrospective	>18	80 TBM
Christopher,et al. 2010(34)	USA	1 January 1993 to 31 December 2005	Retrospective	All age	1896TBM
Krishnapriya,et al. 2020(35)	South India	August 2018 to February 2020	Observational		293 TBM
Patel ,et al.2004(36)	S/Africa	1999 through 2002	Retrospective	All age	6762TBM
Ting,et al. 2016(37)	Shaanxi ,China	September 2010 to December 2012	Retrospective	All age	350 TBM
Jingya,et al.2016(38)	southwest China	-	-	11 to 84	401 TBM
Kavitha,et al. 2016(39)	India	May 2013 – April 2014	Prospective	3 months to 70 years	698 TBM
Duc T,et al.2019(40)	America	01/2010 to 12/2017	Retrospective	All age	192 TBM
Egidia,et al.2015(41)	Romania	2004 to 2013	Retrospective	All age	204 TBM
Erdem,et al.2013(42)	Multi-country	2000 to 2012.	Retrospective	All age	506 TBM
Filiz,et al.2011(43)	Turkey	1998 to 2009	Retrospecti	>14	160 TBM

			ve		
Jyothi,et al.2017(44)	India	2009 to 2014	Retrospective	All age	790 TBM
Lidya,et al.2018(45)	Indonesia	2006 to 2016	Cohort	>18	1180 TBM
Miguel,et al.2020(46)	Mexico	January 2015 to March2018	Retrospective	≥18	41 TBM
Nguyen,et al.2014(47)	Vietnam	17 April 2011 to 31 December 2012	Retrospective	>18	379 TBM
Syed,et al.2017(48)	India	2013 to 2015	-	>18	267 TBM

Table. 2 Sub group analysis of Mortality

Characteristic	Number of studies	Number of deaths	Proportion of death (95%CI)
**Age**			
<18 years	3	95	9.80 (3.22-16.37)
≥18 years	7	277	24.82 (17.05-32.59)
**Study type**			
Retrospective	17	1076	20.34 (14.03-26.65)
other study design	4	160	30.92(18.40-43.44)
**HIV status**			
Positive*	4	220	53.39 (40.55-66.24)
Negative*	4	173	21.65 (4.27-39.03)

Note: *primary studies conducted mortality rate among HIV positive were Jaime,et al.2019; Christopher,et al. 2010; Cecchini,et al.2009 and Fiona,et al.2020.

*Primary studies conducted mortality rate among HIV negative were: Jaime,et al.2019; Christopher,et al. 2010; Cecchini,et al.2009 and Jingya,et al.2016

Supplementary Information

Supplementary Table 1. PRISMA checklist for systematic review and meta-analysis

Section and Topic	Item #	Checklist item	The location where item is reported
**TITLE**			
Title	1	Identify the report as a systematic review.	i
**ABSTRACT**			
Abstract	2	See the PRISMA 2020 for Abstracts checklist.	ii
**INTRODUCTION**			
Rationale	3	Describe the rationale for the review in the context of existing knowledge.	1
Objectives	4	Provide an explicit statement of the objective(s) or question(s) the review addresses.	2
**METHODS**			
Eligibility criteria	5	Specify the inclusion and exclusion criteria for the review and how studies were grouped for the syntheses.	3
Information sources	6	Specify all databases, registers, websites, organisations, reference lists and other sources searched or consulted to identify studies. Specify the date when each source was last searched or consulted.	2
Search strategy	7	Present the full search strategies for all databases, registers and websites, including any filters and limits used.	2
Selection process	8	Specify the methods used to decide whether a study met the inclusion criteria of the review, including how many reviewers screened each record and each report retrieved, whether they worked independently, and if applicable, details of automation tools used in the process.	2
Data collection process	9	Specify the methods used to collect data from reports, including how many reviewers collected data from each report, whether they worked independently, any processes for obtaining or confirming data from study investigators, and if applicable, details of automation tools used in the process.	3
Data items	10a	List and define all outcomes for which data were	3

**Section and Topic**	**Item #**	**Checklist item**	**The location where item is reported**
		sought. Specify whether all results that were compatible with each outcome domain in each study were sought (e.g. for all measures, time points, analyses), and if not, the methods used to decide which results to collect.	
	10b	List and define all other variables for which data were sought (e.g. participant and intervention characteristics, funding sources). Describe any assumptions made about any missing or unclear information.	3
Study risk of bias assessment	11	Specify the methods used to assess risk of bias in the included studies, including details of the tool(s) used, how many reviewers assessed each study and whether they worked independently, and if applicable, details of automation tools used in the process.	4
Effect measures	12	Specify for each outcome the effect measure(s) (e.g. risk ratio, mean difference) used in the synthesis or presentation of results.	4
Synthesis methods	13a	Describe the processes used to decide which studies were eligible for each synthesis (e.g. tabulating the study intervention characteristics and comparing against the planned groups for each synthesis (item #5)).	4
	13b	Describe any methods required to prepare the data for presentation or synthesis, such as handling of missing summary statistics, or data conversions.	4
	13c	Describe any methods used to tabulate or visually display results of individual studies and syntheses.	3
	13d	Describe any methods used to synthesize results and provide a rationale for the choice(s). If meta-analysis was performed, describe the model(s), method(s) to identify the presence and extent of statistical heterogeneity, and software package(s) used.	4
	13e	Describe any methods used to explore possible causes of heterogeneity among study results (e.g. subgroup analysis, meta-regression).	4
	13f	Describe any sensitivity analyses conducted to assess robustness of the synthesized results.	4
Reporting bias	14	Describe any methods used to assess risk of bias due	4

Section and Topic	Item #	Checklist item	The location where item is reported
assessment		to missing results in a synthesis (arising from reporting biases).	
Certainty assessment	15	Describe any methods used to assess certainty (or confidence) in the body of evidence for an outcome.	4
**RESULTS**			
Study selection	16a	Describe the results of the search and selection process, from the number of records identified in the search to the number of studies included in the review, ideally using a flow diagram.	4
	16b	Cite studies that might appear to meet the inclusion criteria, but which were excluded, and explain why they were excluded.	5
Study characteristics	17	Cite each included study and present its characteristics.	5
Risk of bias in studies	18	Present assessments of risk of bias for each included study.	6
Results of individual studies	19	For all outcomes, present, for each study: (a) summary statistics for each group (where appropriate) and (b) an effect estimate and its precision (e.g. confidence/credible interval), ideally using structured tables or plots.	N/A
Results of syntheses	20a	For each synthesis, briefly summarise the characteristics and risk of bias among contributing studies.	N/A
	20b	Present results of all statistical syntheses conducted. If meta-analysis was done, present for each the summary estimate and its precision (e.g. confidence/credible interval) and measures of statistical heterogeneity. If comparing groups, describe the direction of the effect.	10
	20c	Present results of all investigations of possible causes of heterogeneity among study results.	10
	20d	Present results of all sensitivity analyses conducted to assess the robustness of the synthesized results.	10
Reporting biases	21	Present assessments of risk of bias due to missing results (arising from reporting biases) for each synthesis assessed.	N/A
Certainty of	22	Present assessments of certainty (or confidence) in the	11

Section and Topic	Item #	Checklist item	The location where item is reported
evidence		body of evidence for each outcome assessed.	
**DISCUSSION**			
Discussion	23a	Provide a general interpretation of the results in the context of other evidence.	15
	23b	Discuss any limitations of the evidence included in the review.	17
	23c	Discuss any limitations of the review processes used.	17
	23d	Discuss implications of the results for practice, policy, and future research.	16
**OTHER INFORMATION**			
Registration and protocol	24a	Provide registration information for the review, including register name and registration number, or state that the review was not registered.	N/A
	24b	Indicate where the review protocol can be accessed, or state that a protocol was not prepared.	Not prepared
	24c	Describe and explain any amendments to information provided at registration or in the protocol.	N/A
Support	25	Describe sources of financial or non-financial support for the review, and the role of the funders or sponsors in the review.	4
Competing interests	26	Declare any competing interests of review authors.	17
Availability of data, code and other materials	27	Report which of the following are publicly available and where they can be found: template data collection forms; data extracted from included studies; data used for all analyses; analytic code; any other materials used in the review.	18

530 Supplementary Table 2.Quality assessment of included studies

S. no	Author year	Q1				Q2				Q3				Q4				Q5				Q6				Q7				Q8				Q9				Overall quality result
		Yes	No	UC	NA	Yes	No	UC	NA	Yes	No	UC	NA	Yes	No	UC	NA	Yes	No	UC	NA	Yes	No	UC	NA	Yes	No	UC	NA	Yes	No	UC	NA	Yes	No	UC	NA	
1	Ali,et al. 2015	/				/				/				/				/					/			/				/				/				88.8%
2	Anne-Sophie, et al.2011	/				/				/				/				/				/				/					/					/		77.7%
3	Anu ,et al.2018			/		/				/				/				/				/				/				/				/				88.8%
4	Baobao,et al.2021	/					/			/				/				/							/	/				/				/				77.7%
5	Chia,et al. 2017	/				/				/				/				/					/			/				/						/		77.7%
6	Christiene,et al.2002		/			/				/				/				/				/					/			/				/				77.7%
7	Cíntia Helena,et al.2014	/				/				/				/				/				/					/			/				/				88.8%
8	Dong-Mei,et al.2020	/				/				/				/				/				/				/				/				/				100%
9	Fiona,et al.2020	/				/					/			/				/				/				/				/				/				88.8%
10	Gijs,et al.2009	/				/					/			/				/				/					/			/				/				77.7%
11	Heng ,et al.2016	/				/				/				/				/				/				/				/				/				100%
12	Hosoglu ,et al.2003	/				/				/				/				/					/				/			/				/				77.7%
13	Jaime,et al.2019	/				/				/				/				/				/				/				/				/				100%
14	Renu,et al.2017	/				/				/				/				/							/		/			/				/				88.9%
15	Robindra,et al. 2020	/				/				/				/				/				/					/				/			/				66.6%
16	Yahia,et al.2014	/				/				/						/		/					/			/				/				/				66.6%
17	Christopher,et al. 2010	/				/				/				/				/					/			/				/				/				88.8%
18	Krishnapriya,et al. 2020	/				/				/				/				/				/					/			/				/				88.8%
19	Patel ,et al.2004	/				/				/				/				/					/			/				/				/				88.8%
20	Ting,et al. 2016	/				/				/				/				/				/				/					/					/		77.7%
21	Jingya,et al.2016			/		/				/				/				/				/				/				/				/				88.8%
22	Kavitha,et al. 2016	/				/				/				/				/							/	/				/				/				88.8%
23	Duc T,et al.2019	/				/				/				/				/					/			/				/				/				88.8%
24	Egidia,et al.2015		/			/				/				/				/				/				/				/				/				88.8%
25	Erdem,et al.2013	/				/				/				/				/				/					/			/				/				88.8%
26	Filiz,et al.2011	/				/				/				/				/				/				/				/				/				100%
27	Jyothi,et al.2017	/				/					/			/				/				/				/				/				/				88.8%
28	Lidya,et al.2018	/				/					/			/				/				/				/				/				/				88.8%
29	Miguel,et al.2020	/				/				/				/					/				/			/				/				/				77.%
30	Nguyen,et al.2014	/				/				/				/				/					/			/				/				/				88.8%
31	Syed,et al.2017	/				/				/				/				/				/				/				/				/				88.8%

Point-by point response

1. Reference is corrected according to the journal guideline. Please see reference part page 10

2.line 10 - 'studies that reported culture confirmed TBM' this needs to be corrected - the aim was not to search for culture confirmed studies, the aim was to search for presumed/suspected TBM and then estimate the proportion that were culture confirmed

- ✓ Yes the comment is correct and it is corrected as '..studies that reported presumed TBM patients '. See abstract part page 1.

3. Line 33 - 10 million cases of TB were detected - the WHO estimate is of burden (everyone who developed TB), detection is much lower and dropped further due to COVID disruptions

- ✓ This editorial error is corrected as '…the number of people newly diagnosed with TB dropped to 5.8 million..'see page 1

4. Line 68 - add some detail around the expertise and skill required to collect CSF by lumbar puncture (as in your previous response)

- ✓ Some of the skills and requirements for CSF collection are added to the manuscript .see page 2 line 72-74

5.Line 94-95 the definition is not clear - what is this the definition for?

- ✓ Since the operational definition is still ambiguous we want to omit from the manuscript.

6.The discussion requires additional details on the limitations - you may either include these as you discuss the findings or expand the limitations paragraph. These should include:
- trained personnel - for what taking CSF, testing CSF, where is this limited? Any publications that confirm this?

- ✓ In the discussion section we added some discussion on trained personnel and limited skill on taking CSF from lumbar puncture with references. See page 7 line 186-191

- Rate of DR TB - this was only possible in subset of n=XX studies. Also check was these in a particular region and does this DR TB rate reflect underlying DR TB pattern in that region?

- ✓ The following sentence is added to the discussion part under rate of MDR-TB.'…Since most of the included studies to analyze drug resistance pattern were from Asia (5 from India, 4 from china and 1 from Vietnam), the result reflects drug resistance pattern in that specific region.

- HIV TBM mortality - study not able to stratify by CD4 count or ART use; also comment on the study period, could the high HIV TBM be because it occurred in period prior to widespread ART?

- ✓ Majority of the included studies were done after the initiation of antiretroviral treatment in most of developed and developing countries. See page 8 line 221-223

7. Figures - the heading effect size has not been changed as previously recommended and noted in the responses

- ✓ Sorry for the first response ,now it is corrected .please see each figures in the corresponding pages

8. Table 2: please annotate this table so that the HIV studies are specified. Add a footnote so that we can see which of the 4 studies were HIV+ and which were HIV- . The detail on age and study design is in table 1

- ✓ Footnote is added to Table 2.please see page 21 for details.

9.Minor notes which reflect the need for copyediting and proof reading
- the use of Mycobacterium tuberculosis (Mtb) - this needs consistency across the manuscript - please check nomenclature and adjust (lines 32, 35, 46, 61, 185, 195, 205 etc)

- ✓ All the nomenclatures were corrected according to the comment

-TBM to be used consistently

- ✓ We make all the words consistent thought the document

Incorrect capitalization of words - line 53 Infants; line 72 Absence; line 185 Worldwide, line 197 Smear

Check tenses throughout: line 108 'is' should be was

Language: line 156 'further analysis the drug resistance'; line 176 'than expected'; line 179 'definite diagnostic'; line 180 'were got'; line 181-182; line 189 'makes doubt its use';

Line 139 S/Africa needs to be spelled out; line 195 AF

line 139 continent could be deleted

line 143 'rest studies'

- ✓ Capitalization and grammar is corrected thought the document
