# Supplementary material for: Microbiological diagnosis and mortality of tuberculosis meningitis: Systematic review and meta-analysis
Source: PLoS One. 2023 Feb 16;18(2):e0279203. doi: 10.1371/journal.pone.0279203 (PMC9934382; doi:10.1371/journal.pone.0279203)
Supplement: Attachment — Submitted filename: Response to Reviewer.docx [file pone.0279203.s004.docx]

**Response to Reviewer**

**I. Editor comments**

1. **Lines 129-131 and Figure 1: 1354 studies identified with 147 duplicates removed, this should result in 1207 but the next block is 232**

Yes this is clerical error that through the data bases search we found 1354 studies; 1122 duplicates removed and finally 232 studies undergoes title and abstract screening

2. **Figure 2 - uses a column heading effect size but this is actually the proportion not effect size (same in Fig 3, 4, 5, and 6)**

We accept this comment and really it is the proportion not effect size and corrected on the document

3. **Once you have addressed all the comments, please carefully review the syntax and grammar of your manuscript.**

We tried to correct the syntax and grammar of the manuscript through reading and editing

**II. Reviewers' comments**

**Reviewer #1:**

1. **There is need for an extensive review of the grammatical syntax and spellings**

We tried to correct the syntax and grammar of the manuscript through reading and editing

2. **The section on eligibility criteria should be rewritten for clarity**

The eligibility criteria for the study was rewritten as follows

To be included in this study articles must be: (1) original study on TBM suspected patients; (2) published in the English language without regard to a publication year; 3). having described microbiological diagnosis of tuberculous meningitis based on CSF Mycobacteriological culture result data.

**3. Inaccuracies. It is not conceivable that TBM occurs in people who have had tuberculosis. TBM is active tuberculosis (line 38).**

This sentence was rewritten as

Some patients who have or have had tuberculosis may develop the rare complication known as tuberculous meningitis. Tuberculous meningitis accounts for approximately 1 % of all cases of active tuberculosis

**4.Lines 57 and 59 are contradictory regarding the clinical presentation of TBM- the former suggests the presentation is rapid while the latter suggests a sub-acute presentation**

The reported duration of symptoms of tuberculous meningitis ranges between a single day and 6 months, presenting as acute, sub-acute, or chronic meningitis. In children, Most of the time TBM presents as sub-acute meningitis which makes it difficult to distinguishes from other meningoencephalitis diseases (11).

**5. The study case definition is not clear (line 93 – 94)**

We tried to rewrite the study case definition of microbiological diagnoses as follows.

Microbiological diagnoses of Tuberculosis meningitis confirms the suspicion of the disease and identify the etiologic agent by culture from CSF sample.

**6. In lines 225 and 226 one expects lists but these are not presented**

It is re-corrected in the main manuscript as follows

Although we were unable to pinpoint the source of heterogeneity, the following factors could contribute to publication bias and heterogeneity: 1).We only considered research that was published in English; 2).the smallest sample size of the included studies was 20; and 3).the majority of the studies were retrospective.

**7. The high mortality in HIV seropositive participants was noted. Was this stratified by CD4 count or antiretroviral therapy use?**

No this is not stratified by CD4 due to absence of enough data

**8. What is the role of molecular diagnostics like Xpert Ultra (Shen, 2021) and metagenomic sequencing in the diagnosis of TBM?**

As Xpert Ultra increases the sensitivity of Mycobacterium tuberculosis detection and its specificity is 100%, Xpert Ultra had an excellent diagnostic efficacy for TBM, and it could be the preferred initial test for TBM.(Shen,2021).Additionally it gives the result within 2 hours.

Even though metagenomic sequencing is not easily accessible for low and middle income countries, it has moderate sensitivity and high specificity for the diagnosis of tuberculous meningitis (Yu,2020).

**9. Some elements in the conclusion are not supported by the study results**

The conclusion part rewritten based on the findings of the study on the corrected manuscript.

**Reviewer #2: Introduction**

**1.Line 67. Is the lumbar puncture technique a challenge for clinicians in low= and middle- income countries? As in line 182 & 183.**

Yes in low and middle income country there is shortage of trained physicians to draw quality CSF sample through lumbar puncture. This affects the treatment and definite diagnosis of tuberculosis meningitis

**2Methods
.Literature Search
Line 80. Why was literature search limited to Pubmed, EMBASE and gray literature only?**

Because these were the major databases that will contain studies of our interest.Additionalyy in accessibility of other databases.

**3.Lines 85-87. Good key words.
Selection criteria
Lines 96-99. Only inclusion criteria is explicitly stated here. Exclusion criteria should also be clearly stated here and not just later on e.g. lines 229 -231.**

Studies with incomplete data, studies not used culture technique to diagnose TBM, and review articles, meta-analyses and duplicates were all excluded from the study.

**3.Quality Assessment
Line 114. ...high if >80%, medium if 60-80%, and low if 80%. Is this supposed to read, "and low if <60%."**

Really this is a clerical error and it is corrected on the revised manuscript

**4.Results
Study characteristics
Line 138. What was the rational for excluding studies before 1985?**

Here we did no exclude those studies published before 1985 rather the included studies for systematic review and meta-analysis were published from 1985 to 2020

**5.Sub-group analysis of mortality among TBM patients
Lines 163-168. Good description.**

Thanks for your appreciation

**6.Discussion
Line 174. Could the high prevalence of MDR-TB be related to only part of the studies (14/310 reporting this fnding as in line 153?**

Of 31 included studies only 14 report drug susceptibility result of the TBM isolates. There finding, pooled proportion of MDR-TB from these isolates is 5.19%,which support us to say there was high prevalence of MDR-TB among the confirmed tuberculosis meningitis patients.

**7.Lines 178 & 179. Could empirical treatment as opposed to treatment guided by drug sensitivity testing be partly responsible for mortality in TBM?**

This could be supported by further detail trial study
