# Supplementary material for: Microbiological diagnosis and mortality of tuberculosis meningitis: Systematic review and meta-analysis
Source: PLoS One. 2023 Feb 16;18(2):e0279203. doi: 10.1371/journal.pone.0279203 (PMC9934382; doi:10.1371/journal.pone.0279203)
Supplement: Attachment — Submitted filename: Response to reviewer -R.docx [file pone.0279203.s005.docx]

**Point-by point response**

**1**. Reference is corrected according to the journal guideline. Please see reference part page 10

**2**.line 10 - 'studies that reported culture confirmed TBM' this needs to be corrected - the aim was not to search for culture confirmed studies, the aim was to search for presumed/suspected TBM and then estimate the proportion that were culture confirmed

- Yes the comment is correct and it is corrected as ‘..studies that reported presumed TBM patients ‘. See abstract part page 1.

**3**. Line 33 - 10 million cases of TB were detected - the WHO estimate is of burden (everyone who developed TB), detection is much lower and dropped further due to COVID disruptions

- This editorial error is corrected as ‘…the number of people newly diagnosed with TB dropped to 5.8 million..’see page 1

**4**. Line 68 - add some detail around the expertise and skill required to collect CSF by lumbar puncture (as in your previous response)

- Some of the skills and requirements for CSF collection are added to the manuscript .see page 2 line 72-74

**5**.Line 94-95 the definition is not clear - what is this the definition for?

- Since the operational definition is still ambiguous we want to omit from the manuscript.

**6.**The discussion requires additional details on the limitations - you may either include these as you discuss the findings or expand the limitations paragraph. These should include:
- trained personnel - for what taking CSF, testing CSF, where is this limited? Any publications that confirm this?

- In the discussion section we added some discussion on trained personnel and limited skill on taking CSF from lumbar puncture with references. See page 7 line 186-191

- Rate of DR TB - this was only possible in subset of n=XX studies. Also check was these in a particular region and does this DR TB rate reflect underlying DR TB pattern in that region?

- The following sentence is added to the discussion part under rate of MDR-TB.’…Since most of the included studies to analyze drug resistance pattern were from Asia (5 from India, 4 from china and 1 from Vietnam), the result reflects drug resistance pattern in that specific region.

- HIV TBM mortality - study not able to stratify by CD4 count or ART use; also comment on the study period, could the high HIV TBM be because it occurred in period prior to widespread ART?

- Majority of the included studies were done after the initiation of antiretroviral treatment in most of developed and developing countries. See page 8 line 221-223

**7.** Figures - the heading effect size has not been changed as previously recommended and noted in the responses

- Sorry for the first response ,now it is corrected .please see each figures in the corresponding pages

**8.** Table 2: please annotate this table so that the HIV studies are specified. Add a footnote so that we can see which of the 4 studies were HIV+ and which were HIV- . The detail on age and study design is in table 1

- Footnote is added to Table 2.please see page 21 for details.

**9.**Minor notes which reflect the need for copyediting and proof reading
- the use of Mycobacterium tuberculosis (Mtb) - this needs consistency across the manuscript - please check nomenclature and adjust (lines 32, 35, 46, 61, 185, 195, 205 etc)

- All the nomenclatures were corrected according to the comment

-TBM to be used consistently

- We make all the words consistent thought the document

Incorrect capitalization of words - line 53 Infants; line 72 Absence; line 185 Worldwide, line 197 Smear

Check tenses throughout: line 108 'is' should be was
Language: line 156 'further analysis the drug resistance'; line 176 'than expected'; line 179 'definite diagnostic'; line 180 'were got'; line 181-182; line 189 'makes doubt its use';
Line 139 S/Africa needs to be spelled out; line 195 AF
line 139 continent could be deleted
line 143 'rest studies'

- Capitalization and grammar is corrected thought the document
