# Supplementary material for: Microbiological diagnosis and mortality of tuberculosis meningitis: Systematic review and meta-analysis
Source: PLoS One. 2023 Feb 16;18(2):e0279203. doi: 10.1371/journal.pone.0279203 (PMC9934382; doi:10.1371/journal.pone.0279203)
Supplement: Attachment — Submitted filename: Reviewer comments.docx [file pone.0279203.s006.docx]

Reviewer comments

**Introduction**

Adequate literature review.

**Methods**

Protocol and registration followed. Literature search clearly outlined. Not sure about the adequacy of Pubmed and EMBASE only but authors have addressed that particular comment.

Selection criteria is clear with inclusion and exclusion criteria spelt out.

Data extraction: clearly outlined.

Quality assessment also corrected adequately following reviewer comments.

**Data Analysis**

Adequate analysis done and addressed reviewer comments.

**Results**

Eligibility of studies and study characteristics clearly defined.

Happy with study characteristics.

Microbiological diagnosis -adequate description.

Mortality rate and sub-group analysis is well defined.

**Discussion**

Line 179/180: Should this read “The culture confirmed diagnostic rate reported in this study (29.72%) was lower than that reported (38.9%) in a previous study [49]?

Line 180. 70% instead of three-fourths?

Happy with rest of discussion.
